# Supplementary material for: Temporal dynamics of statistical learning in children’s song contributes to phase entrainment and production of novel information in multiple cultures
Source: Sci Rep. 2023 Oct 23;13:18041. doi: 10.1038/s41598-023-45493-6 (PMC10593840; doi:10.1038/s41598-023-45493-6)
Supplement: Supplementary file 1 — Supplementary Tables. [file 41598_2023_45493_MOESM1_ESM.docx]

**Information of Music Corpus**

**Table 1. Summary of sound corpora**

| **Country** | **Number of tones** | **pieces** | **Conditional entropy** |
| --- | --- | --- | --- |
| English | 160.28(±8.891) | 50 | 1.342133016(±0.039) |
| Germany | 43.0875(±2.039) | 80 | 1.107983553(±0.030) |
| Japan | 53.36486486(±3.663) | 74 | 1.171865629(±0.037) |
| Korea | 44.075(±3.284) | 80 | 1.087699492(±0.048) |
| Spain | 49.6(±2.962) | 80 | 1.184260746(±0.029) |

* Average vales in each country (±SEM)

**Table 2. English songs.** All the English song data and materials are publicly archived at <https://zenodo.org/record/4785016#.Y-dQ5OxByvA>. The file names are as follows.

| en001a |
| --- |
| en002a |
| en003a |
| en004a |
| en005a |
| en006a |
| en007a |
| en008a |
| en009a |
| en010a |
| en011a |
| en012a |
| en013a |
| en014a |
| en015a |
| en016a |
| en017a |
| en018a |
| en019a |
| en020a |
| en021a |
| en022a |
| en023a |
| en024a |
| en025a |
| en026a |
| en027a |
| en028a |
| en029a |
| en030a |
| en031a |
| en032a |
| en033a |
| en034a |
| en035a |
| en036a |
| en037a |
| en038a |
| en039a |
| en040a |
| en041a |
| en042a |
| en043a |
| en044a |
| en045a |
| en046a |
| en047a |
| en048a |
| en049a |
| en050a |

**Table 3. German songs.** All the German song data and materials are publicly archived at several websites. The MIDI file cannot be uploaded due to copyright restrictions, but the song titles are as follows.

| a_b_c_die_katze_lief_in_schnee |
| --- |
| abends_wilich_schla |
| alles_schweiget_kanon |
| baeh_laemmel_baeh |
| bruederchen_komm_tanz |
| das_ist_kurz_und_das_ist_lang |
| der_mond_der_scheint |
| droben_auf_gruener_waldheid |
| eia_wiwi_wer_schlaeft_heut_nacht_bei_mir |
| ein_schneider |
| eins_zwei_drei_vier_finkelstein |
| es_regnet_auf_der_bruecke |
| froehliche_weihnacht |
| gut_nacht_mein_feines |
| gut_nacht_mein_liebes_kind |
| guten_abend_gut_nacht |
| guter_mond_du_gehst_s |
| haeschen_in_der_grube |
| hejo_spann_den_wagen_an |
| heut_war_ein_schoener_tag |
| hoppe_hoppe_reiter |
| ich_hab_mir_mein_kindchen |
| ich_hab_mir_mein_kindel_fein_schlafen_gelegt |
| ihr_taeubchen_kommt_alle_zu_mir |
| ist_der_abend_gekommen |
| jakob_hat_kein_brot_im_haus |
| joseph |
| kinderlein_mein |
| kinderwacht |
| kindlein_mein |
| komm_wir_wollen_wandern |
| kommt_gezogen_kleine |
| kuckuck_kuckuck_rufts_aus_dem_wald |
| lange_lange_lise |
| leise_kindelein_leise |
| liebliches_kind |
| lirum_larum_loeffelstiel |
| macht_auf_das_thor |
| mariae_wiegenlied |
| meine_oma_faehrt_im |
| muede_bin_ich_geh_zur |
| muss_wandern_muss_wandern |
| nina_s_fahret_a_bierele_d_rhin_na |
| nun_gebt_uns_auch_ein_peust_ei |
| nun_schlaf_mein_liebes_kindelein 2 |
| o_wie_wohist_mir_am_kanon |
| pipken_pipken_sape |
| puppenwiegenlied |
| reihe_reihe_rose |
| ringle_ringle_reihe |
| rothe_kirschen_ess_ich_gern |
| s_ist_ein_mann_in_brunn_gefalln |
| sandmaennchen |
| sause_kindchen_sause |
| sause_liebe_ninne_was_raschelt_im_stroh |
| schlaf_herzenssoehnch |
| schlafe_kindlein |
| schloap_kindken_profoss |
| singet_leise_leise |
| sinte_martens_vogeltje |
| so_schlaf_in_ruh |
| sonne_hat_sich_mued_gelaufen |
| stille_stille_kein_geraeusch_gemacht |
| stilstilstilwei |
| summ_summ_summ |
| suse_liebe_suse |
| tanze_tanze_telemann |
| toenneken_toenneken_op_den_drag |
| tuarl_haut_gsagt |
| und_als_an_gruener_seite |
| victoria_victoria |
| voeglein_fliegt_dem_n |
| wenn_fromme_kindlein |
| wiegenlied_der_hirten |
| wir_kommen_daher_oh_allen_spott |
| wisst_ihr_wie_die |
| wo_bist_du_denn_gewesen |
| wolln_die_weisen_frauen_fragen |
| zehn_gaens_im_haberstroh |
| zwei_feine_stieflein |

**Table 4. Japanese songs.** All the Japanese song data and materials are publicly archived at several websites. The MIDI file cannot be uploaded due to copyright restrictions, but the song titles are as follows.

| L_aiai |
| --- |
| L_dongurikorokoro |
| L_edono_komoriuta |
| L_furusato |
| L_genkotuyama |
| L_inunoomawarisan |
| L_kaeru_no_gaxtusho |
| L_katatsumuri |
| L_koinobori |
| L_koyamanokousagi |
| L_momiji |
| L_musinokoe |
| L_omotya_no_tyatyatya |
| L_sanpo |
| L_shyabondama |
| L_takeda_no_komoriuta |
| L_tonbo |
| L_toryanse |
| L_tuki |
| L_tyuriztupu |
| L_umi |
| L_uresiihinamatsuri |
| L_usagi_to_kame |
| L_yuki |
| L_yurikago |
| L_zousan |
| L_zuizui |
| P_agarime |
| P_antagatdokosa |
| P_arigatou |
| P_atamadeakusyu |
| P_chatsubo |
| P_chokichokitokoya |
| P_curryrice |
| P_darumasan |
| P_ginganofune |
| P_gutyokipa |
| P_hayakutikotobanouta |
| P_hikarikirakira |
| P_hotaru |
| P_ipponbashikochokocho |
| P_itomaki_no_uta |
| P_kaerusan |
| P_kakurenbo |
| P_kamotsuresha |
| P_kappagawarau |
| P_karasu |
| P_kemushigahiki |
| P_kitune |
| P_kodomotokodomo |
| P_konpirafunefune |
| P_michi |
| P_minnagaatumatta |
| P_motituki |
| P_nabenabe |
| P_nanatsunoko |
| P_ninnokodomo |
| P_ocharaka |
| P_ohoshisamahitotsu |
| P_ojiichanobaachanmo |
| P_ookina_kino_shitade |
| P_osetidezyanken |
| P_oshogatsu |
| P_otanzyoubinonakama |
| P_otentousamanookagedesu |
| P_sakura |
| P_suujinouta |
| P_syupopo |
| P_takibi |
| P_teruteru |
| P_watashinohana |
| P_yokimireba |
| P_yuyake_koyake |
| P_zyuugoyasannomotituki |

**Table 5. English songs.** The 40 Korean song data and materials are publicly archived at <https://zenodo.org/record/4785016#.Y-dQ5OxByvA>. The 40 Korean song data and materials are publicly archived at several websites. The MIDI file cannot be uploaded due to copyright restrictions, but the song titles are as follows (kr011a-kr050a).

| L_chakan_agi_changga |
| --- |
| L_pongnama_changga |
| P_ajossi |
| P_ajukkaridongdong |
| P_anjatta_sotta |
| P_appa_tara |
| P_chamkae |
| P_chamsaeya |
| P_changgulle_ttaettae |
| P_chiickchiick |
| P_hakktoya_changa |
| P_han_tari_tu_tari |
| P_ilbon_taepan |
| P_kamja_kamja |
| P_kangam_tal |
| P_karangnip |
| P_kimchi |
| P_kkokkkoksummora |
| P_kochinsan |
| P_kong_hana_pat_hana |
| P_kongcchinnun_panga |
| P_kyongsaniodego |
| P_molja_molja |
| P_musunpong |
| P_olruk_tolruk |
| P_omma_changga |
| P_onul_haennun |
| P_pan_tal |
| P_panga_panga |
| P_podul_piri_changga |
| P_pom_na_tu_ri |
| P_pomiwannae |
| P_ppalgan_tonggrami |
| P_tal_tta_ro_ka_cha |
| P_urijipyopcchibae |
| P_usumgori |
| P_yennal_yennale |
| P_yennal_yetcchoge |
| P_yopchon |
| P_yukktaegaeju |
| kr011a |
| kr012a |
| kr013a |
| kr014a |
| kr015a |
| kr016a |
| kr017a |
| kr018a |
| kr019a |
| kr020a |
| kr021a |
| kr022a |
| kr023a |
| kr024a |
| kr025a |
| kr026a |
| kr027a |
| kr028a |
| kr029a |
| kr030a |
| kr031a |
| kr032a |
| kr033a |
| kr034a |
| kr035a |
| kr036a |
| kr037a |
| kr038a |
| kr039a |
| kr040a |
| kr041a |
| kr042a |
| kr043a |
| kr044a |
| kr045a |
| kr046a |
| kr047a |
| kr048a |
| kr049a |
| kr050a |

**Table 6. Spanish songs.** All the Spanish song data and materials are publicly archived at several websites. The MIDI file cannot be uploaded due to copyright restrictions, but the song titles are as follows.

| L_a_las_puertas_del_cielo |
| --- |
| L_duermete |
| L_este_nino_lindo |
| L_pajarito_que_cantas |
| L_que_linda_manita |
| L_santana |
| P_a_atocha_va_una_nina |
| P_a_la_rueda_de_san_miguel |
| P_a_la_vibora |
| P_a_la_zapatilla_por_detras |
| P_a_mi_burro |
| P_ahora_que_vamos_despacio |
| P_al_pasar_la_barca |
| P_al_pasar_por_el_cuartel |
| P_aliron |
| P_altaluna |
| P_ambo_ato |
| P_anton_pirulero |
| P_arre_borriquito |
| P_arroz_con_leche |
| P_ay_del_chiquirritin |
| P_brinca_la_tablita |
| P_campana_sobre_campana |
| P_chirimbo |
| P_chocolat |
| P_chocolate_cojita |
| P_cinco_lobitos |
| P_cocherit |
| P_copatata |
| P_dale_dale_la_pinata |
| P_de_mexico_ha_venido |
| P_debboton |
| P_decolores |
| P_diez_perritos |
| P_doncellas_del_prado |
| P_dongato |
| P_el_gato_grande |
| P_el_zopilote |
| P_en_el_balcon_de_palacio |
| P_en_el_salon_del_prado |
| P_eran_tres_alpinos |
| P_escondit |
| P_estaba_una_pastora |
| P_fumfum |
| P_gustablanco |
| P_hacia_belen_va_una_burra |
| P_jose_se_llamaba_el_padre |
| P_la_cucaracha |
| P_la_lluvia |
| P_la_rana_cucu |
| P_la_reina_berenguela |
| P_lolita |
| P_mambru |
| P_mi_granja |
| P_mibarba |
| P_munequita |
| P_nana_caliche |
| P_naranja_dulce2 |
| P_ninamaria |
| P_palmas |
| P_pase_misi_pase_misa |
| P_patiocas |
| P_pavito |
| P_pedcalvo |
| P_perro |
| P_pinpon |
| P_pollitos |
| P_posadas |
| P_quellueva |
| P_reinamar |
| P_san_serenin |
| P_tia_monica |
| P_todos_los_patitos |
| P_tortita_de_manteca |
| P_un_elefante |
| P_un_ratoncito |
| P_vaca_lechera |
| P_ya_se_murio_el_burro |
| P_ya_viene_el_cartero |
| P_yo_te_dare |
